# Investigating Relationship between Pre- and Post- Chemotherapy Cognitive Performance with Levels of Depression and Anxiety in Breast Cancer Patients: A Cross-Sectional Study 

**DOI:** 10.31557/APJCP.2019.20.12.3831

**Published:** 2019

**Authors:** Mohammadreza Hormozi, Seyed Mehdi Hashemi, Sara Shahraki

**Affiliations:** 1 *Department of Psychiatry, *; 2 *Clinical Immunology Research Center, Department of Internal Medicine, Hematology and Medical Oncology Ward, Ali-Ebne-Abitalelb Hospital, *; 3 *GP, Zahedan University of Medical Sciences, Zahedan, Iran. *

**Keywords:** Breast neoplasms, chemotherapy, cognitive dysfunction, cross, sectional studies

## Abstract

**Introduction:**

Evidence suggests that cancer and chemotherapy-related cognitive impairments are an important clinical issue that can have a negative impact on the quality of life (QOL) of many cancer patients during and after treatment. The aim of the current study was to investigate the relationship between cognitive performance before and after chemotherapy with levels of depression and anxiety in patients with breast cancer.

**Materials and Methods:**

This cross-sectional descriptive-analytical study was performed on 100 women with breast cancer in south of Iran. Patients included in the study were evaluated for cognitive performance before chemotherapy and 1, 3, and 6 months after chemotherapy. Patients’ cognitive performance was assessed by Mini-Mental State Examination (MMSE). Patients were also assessed for their level of anxiety and depression using Beck Depression Inventory (BDI) and Beck Anxiety Inventory (BAI). Descriptive tests (percentage, frequency and mean) and ANOVA test used for statistical analysis.

**Results:**

Results of ANOVA test showed a significant difference between the cognitive performance of patients with breast cancer at 1, 3, and 6 months after chemotherapy compared to pre- chemotherapy phase. The above test also revealed a significant relationship between cognitive performance of patients and anxiety and depression.

**Conclusion:**

The results showed that, due to the decrease in cognitive performance and increased anxiety and depression after initiation of chemotherapy in patients with breast cancer, it is necessary to closely monitor the mental and psychological status of these patients by their family and the treatment staffs so that the patient be able to cope with the disease more optimally and to recover.

## Introduction

Brest cancer is the most common type cancer among women and second common type of cancer among all genders that only in 2019, 62,930 new cases of female breast carcinoma were diagnosed (Siegel et al., 2019). Improvements in the treatment process of many cancers have increased the survival time of cancer patients, and researchers have always sought to evaluate and improve QOL of survivors (Ghislain et al., 2016; Bahrieni et al., 2017; Jokar et al., 2017). Many cancer patients have reported impairments such as attention deficit disorder, memory loss, and confused thoughts during and shortly after chemotherapy. Besides, approximately 70% of patients experience these cognitive problems even long after chemotherapy (van Dam et al., 1998; Castellon et al., 2004; Vardy et al., 2007b) , and these impairments may adversely affect daily performance of some patients (Boykoff et al., 2009). Focused research and qualitative interviews with breast cancer patients after chemotherapy show that many of these women are no longer able to carry out their former skills and often lack confidence in returning to their careers (Munir et al., 2011). Survivors also often describe social situations as daunting and may suffer from interpersonal relationships. The chemotherapy-induced cognitive problems clearly lead to a significant decrease in QOL of these patients (Boykoff et al., 2009) .

Studies that have measured cognitive performance using standard neuropsychological assessments have reported mild to moderate effects of chemotherapy on cognitive performance in 15-50% of survivors after the treatment (Vardy and Tannock, 2007; Joly et al., 2011) . Longitudinal studies have shown that cognitive problems in cancer patients can persist for 1 to 2 years after completion of chemotherapy (Schagen et al., 2006; Ahles et al., 2010) and cross-sectional studies have reported cognitive impairments 4 to 10 years after chemotherapy (Kreukels et al., 2006; de Ruiter et al., 2011) . Although most studies have investigated cognitive performance in breast cancer patients, recent studies have shown similar effects in patients with other types of cancer; for example, Vardy et al. found that colorectal cancer patients experience cognitive impairment for up to 6 months after chemotherapy (Vardy et al., 2007a) .

When cognitive impairments (CDs) occur, symptoms are usually seen in processing speed, attention / focus, and executive function, all of which are broadly related to the performance of the frontal lobes of the brain and visual and verbal memory that seems to be controlled by hippocampus controls (Vardy and Tannock, 2007) . In support of this pattern of cognitive disorder, several imaging studies have shown that the chemotherapy-related decrease in white matter integrity is associated with changes in the processing speed of patients (Loh et al., 2016). Other studies have reported changes in electrophysiological indices of processing speed in these patients (Kreukels et al., 2006). Inagaki et al., (2007) have reported brain structural changes in cancer patients 4 months after chemotherapy, which was associated with concentration-attention disorder and memory impairment. Reports of cerebral functional changes have also been reported; De Ruiter et al., (2011)observed hypo-responsiveness in both the prefrontal cortex and the parahippocampal gyrus in cancer survivors who had been undergoing chemotherapy for almost 10 years. Overall, imaging studies have found both structural and functional changes in brain areas which, given these findings, cognitive impairment seem to be experienced by cancer survivors receiving chemotherapy. The issue seems to be somewhat complex, however, as some studies have shown contradictory findings; for example, Shaffer et al., (2012) showed no evidence of a relationship between chemotherapy and cognitive decline in patients with colorectal cancer. Besides, some relevant prospective studies have recently shown that approximately 20% of cancer patients experience cognitive impairment even before chemotherapy (Ahles et al., 2008; Wefel et al., 2010). This suggests that cancer itself and / or cancer diagnosis may also affect cognitive status. In fact, cancer causes systemic changes that may affect cognitive status. Pre-clinical and clinical studies, for example, have shown elevated serum levels of cytokines in cancer patients; on the other hand, increased levels of cytokines and inflammation have been associated with cognitive impairments (Seruga et al., 2008). Although cancerdiagnosis is associated with increased stress, depression, and anxiety, studies evaluating cognitive performance prior to chemotherapy have shown no association between depression / anxiety and cognitive impairment (Schagen et al., 2006; Ahles et al., 2008). However, some studies have shown a relationship between self-reported cognitive symptoms with increased anxiety and depression (van Dam et al., 1998; Castellon et al., 2004); this suggests that cancer diagnosis and treatment-related stress, though partially, can cause cognitive impairments. The very chemotherapy agents commonly used in the clinic also cause significant cognitive impairment in cancer-free laboratory animals. The pattern of cognitive impairment observed in laboratory animals in these studies is almost identical to the one experienced by patients after chemotherapy. These animal studies have suggested hypotheses about the potential chemotherapy-related neurobiological mechanisms associated with cognitive impairments (Ahles and Saykin, 2007; Seigers and Fardell, 2011). Healthy laboratory animals undergoing chemotherapy exhibited increased cell death in the central nervous system (CNS), increased oxidative stress (Cardoso et al., 2019), increased microglia activity, suppressed hippocampal neurogenesis, a decrease in neurotrophic factor levels (Mustafa et al., 2008), and a decrease in the hippocampal catecholamines (Madhyastha et al., 2002), have been demonstrated compared with baseline values.

Overall, the above evidence suggests that cancer and chemotherapy-related cognitive impairment is an important clinical issue that can adversely affect QOL of many cancer patients during and after treatment. The aim of the present study was to evaluate the pre- and post- chemotherapy cognitive performance in patients with breast cancer referred to Hematology Clinic of Imam Ali Hospital and private clinics in Zahedan in 2018-2019. Investigation of the chemotherapy-induced cognitive impairments in cancer patients in the first step can help us to educate these patients about these problems and perhaps improve their treatment process.

## Materials and Methods

*Design and participants*

This cross-sectional study was performed on 100 breast cancer patients who underwent chemotherapy and referred to two educational hospitals in Zahedan, Iran, from October 2018 to March 2019. The STROBE checklist was used to report the study results (Von Elm et al., 2007). Sample size was obtained 86 individuals based on a previous study, which reported pre-and post- chemotherapy cognitive performance in patients with breast cancer as 158.3 ± 27.42 and 142.4±36.42, respectively, with 95% confidence level and test power= 90%. Moreover, considering possible drop-out, 100 individuals were selected using convenience sampling method to increase the accuracy of the study (Janelsins et al., 2017) .

*Eligibility criteria*

All participants were assessed for inclusion criteria. Inclusion criteria included over 18 years of age, being candidate for chemotherapy for breast cancer, having consent to participate in the study. Exclusion criteria also included a history of obvious head trauma (leading to anesthesia for more than an hour or hospitalization), history of marked CNS problems and diseases (Alzheimer’s, Brain mass, meningoencephalitis), CVA history, history of drug use other than chemistry drug that have cognitive complications (benzodiazepines), lack of full consciousness, and a marked sensory problem.

*Instruments*

Data were collected using a four-part instrument. The first part included questions on demographic characteristics including age, gender, type of chemotherapy, and comorbidities. MMSE was used in the second part of the questionnaire to assess the cognitive performance of patients. This standard tool consisted of 20 questions examining cognitive practices in 5 dimensions: orientation, registration, attention, and calculation, recent memory, spatial language and thinking. The total score was 30, with scores of 24 and above, 18-24, and < 18 being considered as a normal cognitive performance, suspected cognitive impairment, and cognitive impairment, respectively. Reliability and validity of this questionnaire have been previously confirmed in a study by Seyednia et al., (2008) in Iran with Cronbach’s Alpha (α=0.81) being calculated for its internal consistency. Beck Depression Inventory (BDI) was used in the third part to assess depression. This standard questionnaire consisted of 21 questions. The possible score range was 0 to 63, with scores 0-13, 14-19, 20-28, and 29-63 indicating minimal, mild, moderate, and severe depression, respectively. Various studies have confirmed its psychometric characteristics (Kaviani et al., 2002; Lashkari-Poor et al., 2008; Nazari et al., 2013) . In the fourth part, Beck Anxiety Inventory (BAI) was used to assess anxiety. This questionnaire is a 21-item self-report tool designed to measure the severity of anxiety syndrome. Each of the items in this questionnaire is assigned scores between 0 and 3. The total BAI score range is 0-63 with scores 0-7, 8-15, 16-25, and 26-63 indicating minimal, mild, moderate, and severe anxiety, respectively. Various studies have confirmed its psychometric characteristics (Kaviani and Mousavi, 2008; Lashkari-Poor et al., 2008; Nazari et al., 2013) .

*Data analysis*

In the present study, the objectives of the study were explained to the participants after coordination with the Chemotherapy Department. Questionnaires were completed by patients before chemotherapy and 1, 3, and 6 months after chemotherapy. Patients were given 20 minutes to complete the questionnaires. Questionnaires were entered into SPSS ver. 18. Descriptive tests (percentage, frequency and mean) were used to describe demographic characteristics. ANOVA test was used to compare the cognitive performance, depression, and patients with breast cancer at 1, 3, and 6 months after chemotherapy with the pre-chemotherapy phase. P< 0.05 was considered as the significance level.

*Ethical considerations*

This study was approved by the Ethics Committee of Zahedan University of Medical Sciences with the number (IR.ZAUMS.REC.1396.170). Written and oral consent was obtained from all participants and they were assured that they could withdraw from the study at any time.

## Results

A total of 100 breast cancer patients were included in the present research. All participants were present at all stages. The mean age of participants was 46.68 ± 9.18 years (range: 32-64). The results showed that the educational level of most of the participants was under-diploma (60%), and most of the patients (72%) had no comorbidities (Table 1)

Results showed that the cognitive performance of patients with breast cancer was significantly decreased during 6 months (p = 0.001). Based on the results of BDI, the pre-chemotherapy depression was at minimal level but the patient had mild depression after the onset of chemotherapy. Based on the results of BAI, patients had moderate anxiety level before and after chemotherapy (Table 2).

Results of ANOVA test showed a significant relationship between cognitive performance of breast cancer patients before and 1, 3, and 6 months after chemotherapy with depression in these patients at the same time periods.

The results of ANOVA test revealed a significant relationship between cognitive performance of breast cancer patients before and 1, 3, and 6 months after chemotherapy with anxiety of these patients at the above-mentioned time periods.

## Discussion

Cancer is one of the major health problems in the world that cause numerous personal, family, and social damages in physical, psychological and social dimensions by threatening human health and active life of various age groups. The aim of the present study was to evaluate the pre- and post- chemotherapy cognitive performance in breast cancer patients. Based on the results of MMSE questionnaire, patients had normal cognitive performance before and one month after chemotherapy, but then their cognitive performance was in suspicious range. There was a significant difference between the cognitive performance of patients with breast cancer at 1, 3, and 6 months after chemotherapy compared to pre-chemotherapy phase. Therefore, their post-chemotherapy cognitive performance should be given special attention, monitored, and strengthened.

Based on the results of Beck Depression Inventory (BDI), the minimal depression level and mild depression were observed before after chemotherapy. Also, based on the results of Beck Anxiety Inventory (BAI), the pre- and post- chemotherapy anxiety levels were in the range of moderate anxiety. In their review study of women with breast cancer in Iran, Mohebi-Fard et al., ( 1397) stated that the results of various studies show that 30-80% of people experience varying degrees of depression and 18-82% of them experience anxiety. There was no significant relationship between depression and anxiety with variables such as place of residence, duration of disease, stage of disease, and type of cancer treatment, but it was significantly related with number of children and family history of depression and anxiety. There were contradictory results concerning the relationship between depression and variables such as age, marital status, occupational status, and educational level. There was also no relationship between anxiety with marital status, employment status, and educational level; while it was related to age. Results showed a negative and significant correlation between factors such as optimism, social support, religious coping and life expectancy with anxiety and depression.

The results showed that women with breast cancer suffer from high levels of depression and anxiety. Therefore, effective interventions, such as appropriate training and psychological interventions, are recommended to be started at the time of diagnosis and continue until after treatment out to reduce psychological distress and. It is suggested to investigate effective risk factors of the early incidence of breast cancer and determine the effect of demographic factors on depression and anxiety more accurately.

There was a significant relationship between cognitive performance of patients with breast cancer before and 1, 3, and 6 months after chemotherapy with depression and anxiety. Rahmani conducted a quasi-experimental study (Rahmani, 2016) and tested a randomized controlled trial at baseline after 2-4 months of follow-up with the control group. Twenty-four patients diagnosed with breast cancer were selected from patients referred to the Oncology and Radiotherapy wards of Imam Hossein Hospital in Tehran and were randomly assigned to experimental (n = 12) and control (n = 12) groups. All participants completed demographic questionnaire, Depression Anxiety Stress Scales (DASS), Metacognitions Questionnaire-30 (MCQ-30), Ruminative Thought Style Questionnaire (RTSQin four stages. In order to comply with ethical issues after obtaining consent from the hospital, the written consent was obtained from all patients. The data were analyzed by multivariate analysis of covariance (MANCOVA). The findings showed that there was a significant difference between experimental and control groups in post-test and 2-4-month follow-up in terms of decreasing symptoms of depression, anxiety, stress, metacognitive beliefs and rumination in women with breast cancer. In another study conducted by Kumar in (2018) to investigate the common differences and severity of symptoms at the start of chemotherapy; and to identify quality of life at 1 year in breast cancer patients, symptoms were seen in 219 pre-breast cancer patients. At baseline, 30 days after the last chemotherapy and 1 year after the first chemotherapy was identified. Patient anxiety and depression scales and experienced symptoms and quality of life were used. Exploratory factor analysis (EFA) was performed to identify symptom clusters at each time point, and then the clusters were compared over time. The prevalence and severity of symptoms have gradually decreased over time.

Long et al., (2016) examined cognitive performance in older adults with primary breast cancer. Participants were initially diagnosed with breast cancer patients who were over 65 years of age without prior systemic treatment or neurological or psychiatric illness and matched the healthy control group. They underwent two assessments: Pre-chemotherapy+ adjuvant therapy and after the end of chemotherapy (doxorubicin + docetaxel) or radiation therapy for patients who did not receive chemotherapy and neuropsychological and aging assessments for healthy control at the same time. Fourth-nine percent of patients (mean age of 70 ± 4 years) were mainly exposed to working memory after cognitive interventions. A total of 64% of these patients developed cognitive impairment after treatment. There was no association with a decline in cognitive problems. There was no significant difference in objective cognitive decline between the two groups of patients. However, the control group had more subjective cognitive complaints after treatment (p = 0.008). It was the largest published study evaluating cognitive performance in older adults with breast cancer, including a group of patients treated with modern chemotherapy regimens. Approximately half of patients were targeted after treatment with cognitive complications.

 Yang et al., (2018) stated that there are approximately 3.1 million breast cancer women living in the United States, with up to 75% of these women having cancer-related cognitive impairment (CRCI). CRCI has been used as a memory disorder, vertigo, a disorder in thought processes and attention. Despite the prevalence of breast cancer, there are only few studies on CRCI, and most of these studies focus mainly on its pathophysiological mechanism. However, recent evidence has shown that breast cancer patients with CRCI are more likely to develop severe psychological depression, suggesting an association between CRCI and psychological distress. A total of 1,498 articles were searched using PubMed, CINAHL, and PsycINFO. Thirteen studies met the inclusion criteria, and one article was removed from the article’s reference list. From these 19-year studies, common psychological symptoms were included in anxiety (n = 3), depression (n = 2), anxiety and depression (n = 4), stress (n = 4), concern (n = 2), mental fatigue (n = 1), and unknown mental disorder (n = 2). Except for six studies designed as a longitudinal study, other studies use a cross-sectional design. Twelve studies used both subjective and objective methods to assess cognitive performance. Patients with severe mental disorder showed lower performance on cognitive performance tests. The findings show that psychological variables contribute to CRCI experienced by breast cancer patients. Areas for further research, which promotes care of breast cancer patients with CRCI, are suggested (Yang and Hendrix, 2018). However, further research needs to be carried out due to the lack of similar studies. One of the most important limitation of study was low sample size of study which prevent of generalization of results. 

In conclusion, The results showed that considering the decline in cognitive performance and increased anxiety and depression after initiation of chemotherapy in breast cancer patients, it is necessary to closely monitor the mental state of these patients by their family and treatment staffs so that the patient can cope with the disease and recover more optimally.

**Table 1:** Demographic Characteristics of Participants in the Two Groups

Variables	Mean± SD
Age	46.68±9.18
	N (%)
Education	N=100
Under diploma	60 (60%)
Diploma	20 (20%)
Bachelor	20 (20%)
Comorbidity	
Hypertension	12 (12%)
Diabetes	16 (16%)
No comorbidity	72 (72%)

**Table 2:** Determination of Cognitive Performance, Depression, and Anxiety in Patients with Breast Cancer Before and 1, 3, and 6 Months after Chemotherapy

Variables	Mean± SD	P-value
Cognitive performance		
Before chemotherapy	25.20±4.02	
After one month	24.92±3.36	0.001
After three months	23.80±4.04	
After six months	22.80±4.22	
Depression		
Before chemotherapy	12.96±9.95	
After one month	14.44±12.08	0.02
After three months	16.12±10.97	
After six months	19.16±13.16	
Before chemotherapy	16.52±11.07	
Anxiety		
After one month	15.32±11.54	0.02
After three months	16.80±11.80	
After six months	20.88±13.50	

**Table 3:** Determining the Relationship between Depression and Anxiety with Cognitive Problems in Breast Cancer Patients

Variables	F	P- value
Pre-chemotherapy depression and pre-chemotherapy cognitive performance	16/94	<0/001
Depression 1 month after chemotherapy and pre-chemotherapy cognitive performance	7/57	<0/001
Depression 3 months after chemotherapy and pre-chemotherapy cognitive performance	7/92	<0/001
Depression 6 months after chemotherapy and pre-chemotherapy cognitive performance	9/32	<0/001
Pre-chemotherapy depression and cognitive function 1 month after chemotherapy	5/53	<0/001
Depression 1 month after chemotherapy and cognitive performance 1 month after chemotherapy	6/82	<0/001
Depression 3 months after chemotherapy and cognitive performance 1 month after chemotherapy	11/10	<0/001
Depression 6 months after chemotherapy and cognitive performance 1 month after chemotherapy	7/50	<0/001
Pre-chemotherapy depression and cognitive performance 3 months after chemotherapy	11/55	<0/001
Depression 1 month after chemotherapy and cognitive performance 3 months after chemotherapy	20/61	<0/001
Depression 3 months after chemotherapy and cognitive performance 3 months after chemotherapy	18/71	<0/001
Depression 6 months after chemotherapy and cognitive performance 3 months after chemotherapy	9/25	<0/001
Pre-chemotherapy depression and cognitive performance 6 months after chemotherapy	11/76	<0/001
Depression 1 month after chemotherapy and cognitive performance 6 months after chemotherapy	17/88	<0/001
Depression 3 months after chemotherapy and cognitive performance 6 months after chemotherapy	18/04	<0/001
Depression 6 months after chemotherapy and cognitive performance 6 months after chemotherapy	11/48	<0/001

**Table 4:** Determining the Relationship between Anxiety and Cognitive Problems in Breast Cancer Patients Based on ANOVA Test

Variables	F	P- value
Pre-chemotherapy depression and pre-chemotherapy cognitive performance	11/51	<0/001
Depression 1 month after chemotherapy and pre-chemotherapy cognitive performance	10/94	<0/001
Depression 3 months after chemotherapyand pre-chemotherapy cognitive performance	14/50	<0/001
Depression 6 months after chemotherapy and pre-chemotherapy cognitive performance	14/61	<0/001
Pre-chemotherapy depression and cognitive function 1 month after chemotherapy	6/55	<0/001
Depression 1 month after chemotherapy and cognitive performance 1 month after chemotherapy	7/27	<0/001
Depression 3 months after chemotherapy and cognitive performance 1 month after chemotherapy	8/10	<0/001
Depression 6 months after chemotherapy and cognitive performance 1 month after chemotherapy	14/14	<0/001
Pre-chemotherapy depression and cognitive performance 3 months after chemotherapy	12/19	<0/001
Depression 1 month after chemotherapy and cognitive performance 3 months after chemotherapy	16/60	<0/001
Depression 3 months after chemotherapy and cognitive performance 3 months after chemotherapy	15/41	<0/001
Depression 6 months after chemotherapy and cognitive performance 3 months after chemotherapy	4/70	<0/001
Pre-chemotherapy depression and cognitive performance 6 months after chemotherapy	14/08	<0/001
Depression 1 month after chemotherapy and cognitive performance 6 months after chemotherapy	29/43	<0/001
Depression 3 months after chemotherapy and cognitive performance 6 months after chemotherapy	29/20	<0/001
Depression 6 months after chemotherapy and cognitive performance 6 months after chemotherapy	36/55	<0/001
